# Role of a GenoType MTBDR*plus* line probe assay in early detection of multidrug-resistant tuberculosis at a Brazilian reference center

**DOI:** 10.1590/1414-431X20154458

**Published:** 2015-06-30

**Authors:** C.S. Feliciano, M.M.P. Nascimento, L.M.P. Anselmo, R.H.C. Pocente, F. Bellissimo-Rodrigues, V.R. Bollela

**Affiliations:** 1 Departamento de Clínica Médica, Faculdade de Medicina de Ribeirão Preto, Universidade de São Paulo, Ribeirão Preto, SP, Brasil; 2 Departamento de Medicina Social, Faculdade de Medicina de Ribeirão Preto, Universidade de São Paulo, Ribeirão Preto, SP, Brasil

**Keywords:** Multidrug-resistant tuberculosis, Molecular diagnostic techniques, Risk factors

## Abstract

Resistance to *Mycobacterium tuberculosis* is a reality worldwide, and its diagnosis continues to be difficult and time consuming. To face this challenge, the World Health Organization has recommended the use of rapid molecular tests. We evaluated the routine use (once a week) of a line probe assay (Genotype MTBDR*plus*) for early diagnosis of resistance and for assessment of the main related risk factors over 2 years. A total of 170 samples were tested: 15 (8.8%) were resistant, and multidrug resistance was detected in 10 (5.9%). The sensitivity profile took 3 weeks (2 weeks for culture and 1 week for rapid testing). Previous treatment for tuberculosis and the persistence of positive acid-fast smears after 4 months of supervised treatment were the major risk factors observed. The use of molecular tests enabled early diagnosis of drug-resistant bacilli and led to appropriate treatment of the disease. This information has the potential to interrupt the transmission chain of resistant *M. tuberculosis*.

## Introduction

Brazil is among 22 countries that have a concentration of approximately 80% of all tuberculosis (TB) patients, and occupies the 16th position in absolute numbers of cases (1). Similar to other countries, there has been great progress in controlling the disease in Brazil in the last two decades, since TB was declared to be a worldwide public health emergency by the World Health Organization (WHO) (1,2).

Although there have been consistent advances in controlling measures, there is concern about increasing resistance by *Mycobacterium tuberculosis* to anti-TB drugs (1,3). The bacillary resistance is still rarely diagnosed, mainly due to the low access to sensitivity tests in countries with greater incidences of the disease (1,4). This is related to the scarcity of available data on the epidemiology of resistance, as evidenced in Brazil (3,5).

In this regard, the development and application of strategies for allowing a fast and effective diagnosis of these cases have gained international attention (6,7), and since 2008, rapid molecular tests for the detection of resistance to anti-TB drugs have been recommended by WHO (8). Among them is the Genotype MTBDR*plus* (Hain Lifescience, Germany) line probe assay (LPA), approved for use with specimens growing in culture media and also for use with smear-positive sputum samples. This test identifies *M. tuberculosis* complex bacilli and detects mutations in three genes: *rpoB,* which confers resistance to rifampicin; *KatG*, which confers high-level resistance to isoniazid; and the *inhA* regulatory region, which confers low-level resistance to isoniazid (8).

The objective of this study was to evaluate the systematic use of an LPA to diagnose resistance of *M. tuberculosis* and the potential meaning of this information in decision making for patients treated in a tertiary reference center in the State of São Paulo, Brazil. We also evaluated possible risk factors associated with bacillary resistance in these patients.

## Material and Methods

### Study population

All patients at the Hospital das Clínicas, Faculdade de Medicina de Ribeirão Preto, Universidade de São Paulo (USP), who had been diagnosed with tuberculosis by bacillary growth in culture media during 2012 and 2013, were eligible for this study. The project was approved by the Ethics and Research Committee of the Hospital das Clínicas de Ribeirão Preto, USP.

### Data collection

#### Collecting samples

Clinical samples were collected during routine diagnostic investigation of TB, according to the indication of the medical team that cared for the patient, following the routine already established in the mycobacteria laboratory: direct exam after Ziehl-Neelsen staining and incubation in liquid medium culture in the automated system MGIT 960. Identification of the species was performed by a polymerase chain reaction technique that amplifies a fragment of 123 base pairs of the IS6110 region of *M. tuberculosis* (9).

#### LPA

The rapid molecular test Genotype MTBDR*plus* was carried out once a week for all strains isolated in culture during that period, following the manufacturer's instructions (Hain Lifescience). It is a genotypic test that identifies the *M. tuberculosis* complex and detects mutations that confer resistance to rifampicin and isoniazid. The identification of resistance to rifampicin was determined by detection of mutations in the *rpoB* gene, which codifies the RNA polymerase β-subunit. With regard to resistance to isoniazid, we evaluated mutations in the *KatG* gene (that codifies catalase peroxidase), which confers high-level resistance to isoniazid, and in the *inhA* regulatory region (that codifies the enoyl-acyl carrier protein reductase), which confers low-level resistance to isoniazid (8).

The procedure involved extraction, multiplex amplification with biotinylated primers, and DNA reverse hybridization. The result was determined and interpreted on a strip. The positive control uses a probe, which identifies *M. tuberculosis* complex, marked as TUB in the strip. As quality controls, the strip had the following probes: conjugate control (CC), amplification control (AC), and locus control (*rpoB*, *KatG*, and *inhA*). The strip was also composed of wild-type (WT) probes that included the most important resistance areas of the referred genes (*rpoB* WT 1 to 8, *KatG* WT, and *inhA* WT 1 and 2). If they were present, they excluded detectable mutations in both genes and in the regulatory region that was being evaluated. Other components of the strip were mutation probes, which detected some of the most common mutations that cause resistance. Probes that were positive in the locus of a mutation reflected the mutation in the gene or in the regulatory region evaluated. For the *rpoB* gene, we evaluated the mutations D516V, H526Y, H526D, and S531L. For the *KatG* gene, we evaluated mutations S315T1 and S315T2. In relation to the *inhA* regulatory region, we evaluated mutations C15T, A16G, T8C, and T8A. The absence of a WT band or the presence of a mutant band was an indication of resistance to the drug evaluated. For interpretation, the strip was compared with an evaluation form provided by the manufacturer (8,10).

#### Phenotypic sensitivity testing

Samples that were resistant to rifampicin and/or isoniazid in the LPA were evaluated by the reference laboratory for the resistance of mycobacteria through nonradiometric phenotypic sensitivity testing in liquid medium BACTEC MGIT 960 (MGIT 960; Becton Dickinson Diagnostic Systems, USA). The reference laboratory (which is in the capital of São Paulo state) receives and tests only strains from patients suspected of having resistant TB. The time between sending the strains and receiving the resistance results was 6 weeks.

#### Risk factors for resistance

Information on risk factors for resistance was obtained through the review of medical records. We evaluated sociodemographic data, habits (elitism, smoking, and drug addiction), history of treatment, previous therapeutic failure, history of contact with cases of multidrug-resistant tuberculosis (MDR-TB), street population, penitentiary population, infection by human immunodeficiency virus (HIV), and alterations in pulmonary imaging examinations.

### Statistical analysis

Data were analyzed by the Stata statistical software, version 12.0 (StataCorp LP, USA). The study involved the description of the sensitivity profile of *M. tuberculosis* strains. To test the association among the variables studied and the occurrence of resistant tuberculosis (to rifampicin, isoniazid, or both), a two-tailed Fisher's exact test was used.

With the aim of determining which of these variables exhibited real association with the outcome of resistant tuberculosis, a model of logistic regression was built, using as independent variables those showing P≤0.10 in univariate analysis using Fisher's test.

## Results

### Sensitivity profile and patient characterization

Samples from 170 patients diagnosed with TB were included in the period of the study (134 men and 36 women; mean age 41.8 years). The pulmonary form of the disease occurred in 120 patients (70.6%) and the other 50 patients showed disseminated and/or extrapulmonary forms, with the most frequent site being pleura in 15 cases (8.8% of the total).

From the 170 included samples, 155 (91.2%) showed sensitivity to rifampicin and isoniazid, and 15 (8.8%) showed some resistance profile. Multidrug-resistant isolates reached 5.9% (10/170) of total cases, monoresistance to rifampicin 1.2% (2/170), and monoresistance to isoniazid 1.7% (3/170).

Primary MDR-TB (patients without prior TB treatment) occurred in two cases (1.2%) and acquired MDR-TB in eight cases (4.7%). Primary monoresistance to isoniazid was detected in one patient (0.6%) and acquired was detected in two patients (1.2%). Primary monoresistance to rifampicin did not occur in this sample, and the acquired resistance to this drug was detected in two patients (1.2%).

**Figure 1:**
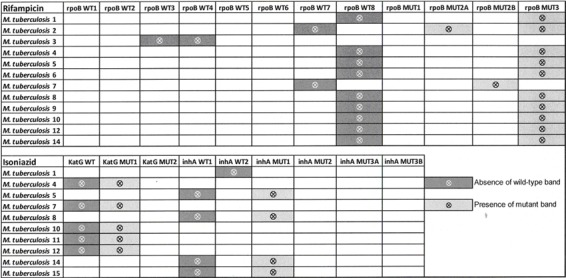
Schematic representation of the Genotype MTBDR*plus* results in isolates resistant to rifampicin and/or isoniazid.

From 12 samples that exhibited resistance to rifampicin by the molecular test Genotype MTBDR*plus*, 9 (75%) showed the same genotypic profile of resistance, i.e., loss of the wild-type band WT8 and appearance of the MUT3 band of the *rpoB* gene, which infers the mutation S531L. The genotypic profile of resistance to isoniazid appeared to be very heterogeneous in this sample: five isolates had a high-level resistance profile to the drug (loss of wild-type band and appearance of the band referring to MUT1 of the *KatG* gene, which infers the mutation S315T1); and the other five isolates showed a low-level resistance pattern. Of those, one showed a loss of the WT2 wild-type band and four showed a loss of WT1 and presence of the band referring to MUT1 of the *inhA* regulatory region, which infers the mutation C15T (Figure 1).

The mean time for obtaining resistance results of the molecular test was 3 weeks, including the 2 weeks until signaling growth in the liquid medium MGIT-96 and 1 week for the Genotype MTBDR*plus* sensitivity test.

For those samples with both LPA and reference laboratory multidrug-resistant results, there was the same resistance profile (100%) for rifampicin and 80% for isoniazid. In two of these isolates, the LPA did not demonstrate the resistance detected in the phenotypic testing.

### Evaluation of possible risk factors associated with resistance

Among the possible risk factors analyzed, we determined the association of the grouped variables: brown and black color, history of treatment, and previous therapeutic failure, with statistically significant differences.

The history of contact with a MDR-TB carrier seems to have an association with the development of resistance; however, in the present sample only two patients had this risk factor and there was no statistically significant difference. We demonstrated an association between resistant TB and the presence of radiological or tomographic findings of pulmonary fibrosis, with statistically significant differences. The presence of pulmonary caves also demonstrated this association, but without any statistically significant difference (Table 1).

**Table:**
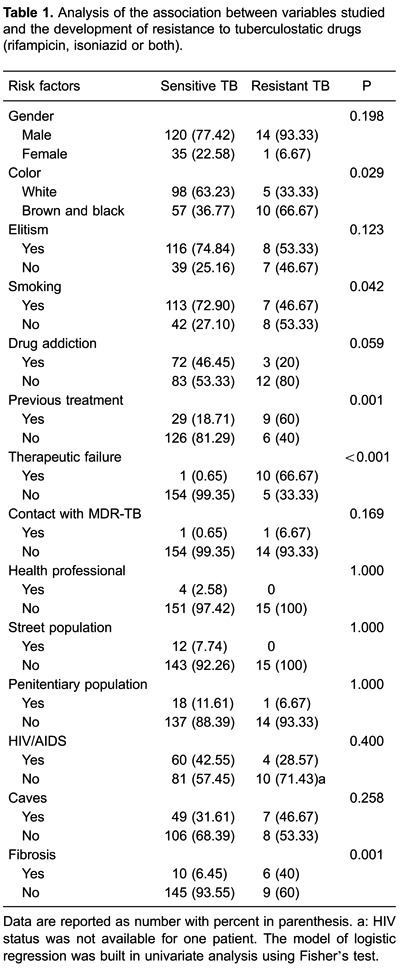

With the logistic regression model, we found that only the variable “therapeutic failure” exhibited a real association with the outcome of resistant disease, with an odds ratio of 103.53 and confidence interval between 9.6 and 1109.5 (Table 2). It is important to point out that there was no positive association in patients with HIV/acquired immune deficiency syndrome (AIDS) in the present study.

**Table:**
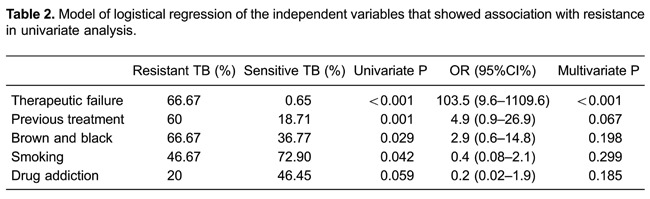

### Follow-up information for the 15 patients with resistant *M. tuberculosis*

From 15 patients with confirmed resistance, 11 (73.3%) were cured and 4 (26.7%) died during the treatment. Among the patients who died, three were also infected with HIV/AIDS (1 MDR-TB, 1 rifampicin resistant, and 1 isoniazid resistant). The fourth patient had previous MDR-TB, treated and cured in 2009/2010 with a relapse in 2011, when *M. tuberculosis* was isolated again and presented with resistance to rifampicin, isoniazid, and ethambutol, without HIV infection.

## Discussion

Of the 170 strains of *M. tuberculosis* analyzed, 155 (91.2%) showed sensitivity both to rifampicin and to isoniazid and 15 (8.8%) showed a resistance profile to both drugs. By evaluating the resistance data in the total sampling, we observed 5.9% (10 cases) with multidrug resistance, 1.2% (2 cases) of monoresistance to rifampicin, and 1.7% (3 cases) of monoresistance to isoniazid. Primary MDR-TB occurred in two (1.2%) cases and acquired MDR-TB in eight (4.7%) cases.

Data are scarce globally and nationally about resistance to the first line of anti-tuberculosis drugs. This fact used to be related to the scarcity of trained personnel and equipped laboratories to conduct sensitivity tests and poor logistics for the shipment of samples that needed to be tested. These factors have hindered, in practice, more regular analyses of the sensitivity profile of *M. tuberculosis*, especially in countries with a high prevalence of TB, MDR-TB, and tuberculosis and HIV co-infection (1,3).

Data from WHO, between 1994 and 2010, estimated the prevalence of primary and acquired MDR-TB as 3.4% and 19.8%, respectively (11). It is estimated that, in the year 2012, the incidence of MDR-TB was 5.2% and it is increasing (1).

Evaluating data from the II Brazilian Inquiry of Resistance to anti-TB drugs, which was carried out between 2007 and 2008, involving 4421 patients, the rate of primary resistance to rifampicin and isoniazid was 1.4% and acquired resistance was 7.5%. Primary monoresistance to isoniazid was 6% and acquired was 15.3%. For rifampicin, primary and acquired resistance were 1.5% and 8%, respectively (3).

In this study, 9 of the 12 samples that showed resistance to rifampicin in the rapid molecular test had the same genotypic profile of resistance (loss of *rpoB* WT8 wild-type band and appearance of *rpoB* MUT3 band), which infers the mutation S531L. Hillemann et al. (12) found similar data in their study, in which the mutation S531L occurred in 73.6% of the samples evaluated by Genotype MTBDR*plus*. Vijdea et al. (10) and Yadav et al. (13) found, respectively, 86% and 72% of the S531L mutation in strains resistant to rifampicin that were subjected to the same genotypic testing.

Different from that observed in the resistance to rifampicin, the genotypic profile to isoniazid resistance was shown to be very heterogeneous in this sample. There were five isolates with high-level resistance profiles to the drug due to mutation S315T1 (*KatG* gene) and five with low-level resistance patterns, and four of them were due to mutation C15T (*inhA* regulatory region).

Vijdea et al. (10) evaluated two distinct subgroups in relation to the resistance profile to isoniazid. In one of the subgroups, the S315T1 mutation was observed in all isolates tested, and in the other group the mutation C15T was the most frequent (86%) among the isolates with low-level resistance to the drug. Lacoma et al. (14) observed that, in isolates with high-level resistance, 80.9% showed the mutation S315T in the *KatG* gene.

Without the LPA, the mean time for clinicians to identify the *M. tuberculosis* sensitivity profile in our hospital is from 8 to 10 weeks, including growth time of the bacillus (2 weeks), transportation to the reference laboratory, and phenotypic sensitivity testing (6-8 weeks). In this study with the LPA incorporated in the mycobacteria laboratory routine, it was possible to obtain the results of sensitivity tests to rifampicin and isoniazid 3 weeks after receiving the sample in the hospital laboratory. This information may have a potential positive impact on the decision-making process for TB patients, mainly by reducing the time to begin correct treatment, as described by other studies in areas with high prevalence of MDR-TB (15,16).

A finding to be highlighted was the detection of resistance with higher frequency in patients with smear-positive samples 4 months after regular treatment of the disease (characterizing initial treatment failure). This finding is a known sensitive indicator for resistant cases in clinical practice, and is easily available, because it can be obtained during follow-up of patients in treatment by performing smear examinations on a monthly basis during the use of TB medication.

Among the possible risk factors associated with resistance are previous treatments for tuberculosis, as has been frequently reported by many authors (17 18 19 20). In this study, previous treatment followed by relapse of the disease was an important risk factor for the development of resistance. This is corroborated by the presence of the highest frequency of radiological alterations of the type pulmonary “fibrosis” among patients who had been treated previously for tuberculosis and cured.

In our study, we did not observe statistically significant differences between the presence of pulmonary cavities and the occurrence of resistance. However, there are some studies demonstrating this association, because both primary and acquired resistances are phenomena dependent on bacillary load and active multiplication, which is much higher in the presence of cavitary disease (21,22).

The association between brown and black colors and *M. tuberculosis* resistance seems to be related more to the poor socioeconomic and housing conditions of these population groups in Brazil, as already observed in another study in the country (21).

There is great divergence in the literature about the role played by HIV infection, and many authors have not found this association (19,21,23,24). Certainly, the number of TB-resistant cases included in this study limited evaluation of an association between HIV infection and the development of resistance by the bacillus to the two main drugs of the anti-TB scheme. This limitation is also valid for the evaluation of other risk factors analyzed.

An important limitation of this study is the fact that the molecular sensitivity test was only performed on bacilli that grew in liquid culture medium and were from patients who do not represent all the TB cases diagnosed in the hospital during the period of the study. There was a group of patients (less than 20 registered as TB cases) who did not have culture confirmation. Most were immunosuppressed patients with severe disease, suspected of TB, from whom it was not possible to isolate the bacillus, so empirical treatment was initiated.

Another limitation was the lack of phenotypic testing on the strains that were sensitive using the LPA. The reference laboratory does not perform analyses for patients without any indication for TB-resistant testing. However, when reviewing the cases, we observed clinical and microbiological cures with conventional treatment for the cases with LPA bacillus sensitivity to rifampicin and isoniazid.

Finally, this study was not designed to compare patient outcomes before and after LPA introduction to the Mycobacteria laboratory routine, but it definitely contributed to the early start of MDR treatment in our setting.

In conclusion, despite consistent advances in the control of tuberculosis, the challenge of increasing resistance to anti-TB drugs persists. Considering the limited number of recent national data about the epidemiology of *M. tuberculosis* resistance, it becomes urgent to build an epidemiological profile of the bacillary resistance in the country. To reach this goal it would be critical to optimize and increase access to sensitivity tests, and the LPA could be an effective and affordable option. With deeper knowledge of the epidemiological resistance profile, in the future it could be possible to define groups of patients at higher risk to host-resistant strains according to the region in which they live. Still, early diagnosis of resistance has the potential to impact the decision for the moment at which to begin treatment and cure, with a possible positive influence on the transmission chain of resistant bacilli.
